# Gestational Diabetes Mellitus and Preeclampsia: Correlation and Influencing Factors

**DOI:** 10.3389/fcvm.2022.831297

**Published:** 2022-02-16

**Authors:** Ying Yang, Na Wu

**Affiliations:** ^1^Department of Gastroenterology, Shengjing Hospital of China Medical University, Shenyang, China; ^2^Department of Endocrinology, Shengjing Hospital of China Medical University, Shenyang, China; ^3^Clinical Skills Practice Teaching Center, Shengjing Hospital of China Medical University, Shenyang, China

**Keywords:** gestational diabetes, preeclampsia, pregnancy, polycystic ovary syndrome, obesity

## Abstract

Gestational diabetes mellitus (GDM) and preeclampsia (PE) are common pregnancy complications with similar risk factors and pathophysiological changes. Evidence from previous studies suggests that the incidence of PE is significantly increased in women with GDM, but whether GDM is independently related to the occurrence of PE has remained controversial. GDM complicated by PE further increases perinatal adverse events with greater impact on the future maternal and offspring health. Identify factors associated with PE in women with GDM women, specifically those that are controllable, is important for improving pregnancy outcomes. This paper provides the findings of a review on the correlation between GDM and PE, factors associated with PE in women with GDM, possible mechanisms, and predictive markers. Most studies concluded that GDM is independently associated with PE in singleton pregnancy, and optimizing the treatment and management of GDM can reduce the incidence of PE, which is very helpful to improve pregnancy outcomes.

## Introduction

Gestational diabetes mellitus (GDM) and preeclampsia (PE) are common complications in pregnancy with similar risk factors, including obesity, advanced age, and multiple pregnancy (1, 2). Moreover, in both GDM and PE, the pathophysiological processes involve oxidative stress, pro-inflammatory factor release, vascular endothelial dysfunction (3, 4), which all increase the risk of future maternal diabetes and cardiovascular disease (5–8); thus, a correlation between GDM and PE may exist.

GDM is defined as glucose intolerance diagnosed for the first time during pregnancy (9). The International Association of Diabetes and Pregnancy Study Groups (IADPSG) recently updated the diagnostic criteria for GDM according to the findings of the Hyperglycemia and Adverse Pregnancy Outcome (HAPO) study, an oral glucose tolerance test (OGTT) must be performed in a fasting state using 75 g of glucose at 24–28 weeks (10). In 2013, the World Health Organization (WHO) further defined the diagnostic criteria of GDM. GDM is defined as meeting the above 75 g OGTT diagnostic criteria at any time during pregnancy, and the upper limit levels of fasting and 2-h blood glucose were defined (11). With the increasing prevalence of obesity and changes in people's lifestyle, the prevalence of GDM has also significantly increased two to three times in ~10 years (12–14). The prevalence rate of GDM in the Middle East and some North African countries has reached 15.2% (2), while that of Chinese mainland is 14.8% (15). Increased insulin resistance and pancreatic β-cell dysfunction are the major pathogenesis of GDM, which may already exist before pregnancy, especially in obese populations (2). GDM is associated with adverse pregnancy outcomes. Studies have found that the incidence of PE is significantly increased in GDM (16, 17). However, whether GDM is independently associated with the occurrence of PE or because of the effects of their common risk factors, especially obesity, remains controversial.

PE refers to new hypertension (systolic or diastolic blood pressure ≥140 or ≥90 mmHg, respectively) diagnosed at or after 20 weeks of gestation with proteinuria, or at least one other organ (kidney, liver, nervous system, blood system, and uteroplacenta) dysfunction (18). PE is the main cause of maternal and fetal mortality and morbidity (19, 20). GDM complicated by PE further increases perinatal adverse events (21–24), future maternal risk of chronic hypertension, cardiovascular disease, and diabetes (25–27); offspring body mass index (BMI) also steadily increases over time (28). Identifying factors associated with occurrence of PE in women with GDM, especially those that are controllable, is important for improving pregnancy outcomes. This review describes the relationship between GDM and PE, factors associated with occurrence of PE in women with GDM, and impact of GDM on PE in twin pregnancy and in pregnant women with polycystic ovary syndrome (PCOS). It also explores possible impact mechanisms and predictive markers to improve pregnancy outcomes.

## Search Strategy and Selection Criteria

We retrieved studies from the PubMed, Ovid, and Wiley from the inception of the databases to June 2021, with the search terms “gestational diabetes mellitus” and “preeclampsia.” We cross-referenced these terms with “obesity,” “body mass index,” “gestational weight gain,” “early onset,” “blood glucose,” “polycystic ovary syndrome,” “twin pregnancy,” “management,” “mechanism,” “predictive markers,” “risk factors,” “insulin,” “metformin,” “Glibenclamide.” We carefully screened all the articles, and focused on articles covering multivariate analysis to judge the independent correlation.

## Correlation Between GDM and PE

HAPO is a large international prospective blinded cohort study involving 23,316 pregnant women in 15 centers from nine countries, assessing the relationship between blood glucose below diabetes levels and pregnancy outcomes. The HAPO study found that the occurrence of PE is positively associated with blood glucose level even after adjusting for clinical center, age, BMI, height, smoking status, alcohol consumption, family history of diabetes, gestational age at OGTT, and urinary tract infection (29). Following the IADPSG diagnostic criteria, secondary analysis showed that non-obese women with GDM was also associated with PE after adjusting for the above confounding factors, but the association was lower than obesity (16). Population-based retrospective cohort studies in several countries also showed GDM was independently associated with the occurrence of PE (17, 30–40). According to a retrospective cohort study in Sweden, obesity is the main confounding factor (32); however, another retrospective cohort study in France suggested that obesity is not related with the occurrence of PE in women with GDM (36). Different diagnostic criteria for GDM have had little impact on the occurrence of PE (41). A few studies suggest that GDM is not associated with the occurrence of PE after removing the effect of pre pregnancy BMI and other factors (42–46). A retrospective cohort study in Germany showed that there was no independent correlation between GDM and PE, regardless of obesity before pregnancy, and it was unknown whether it was related to the strict control of blood glucose levels (45). In studies conducted in Australia and Japan (42, 43), cases included those within the diagnostic criteria of IADPSG but not up to their own national standards, so blood glucose levels were relatively low, which may have affect the results. Based on these previous findings (Table 1), most studies support that GDM was independently associated with the occurrence of PE in singleton pregnancy. In addition, GDM is also a major risk factor for recurrent (47) and new postnatal PE in the absence of a PE history (48). The history of GDM in first pregnancy is also a risk factor for PE in the second pregnancy (49).

**Table 1:** The independent association of gestational diabetes mellitus with preeclampsia.

**Country**	**References**	**Study period**	**Type of birth**	**Study design**	**GDM criteria**	**Number of GDM**	**PE% GDM**	**Study content**	**Result**
						**/No GDM**	**/No GDM**		
Nine countries	Catalano et al. (16)	2000–2006	Single	PC	IADPSG	2,518/16,238	5.9/3.5	Association	Positive
Australia	Stone et al. (37)	1996	Single	RC	NR	2,169/58,231	8.1/5.2	Association	Positive
	Cheung et al. (42)	2014–2016	Single	RC	IADPSG	375/4,873	4.0/2.0	Association	Negative
Brazil	Schmidt et al. (41)	1991–1995	All	PC	ADA2000(75g OGTT)/	Total 4,572	NR	Association	Positive
					WHO1999			Association	Positive
Canada	Nerenberg et al. (34)	2000–2009	All	RC	CDA	15,404/407,268	2.6/1.2	Association	Positive
	Lai et al. (40)	2005–2011	Single	RC	CDA	18,137/306,576	3.4/1.7	Association	Positive
	Hiersch et al. (35)	2012–2016	Single	RC	CDA	16,731/250,211	1.1/0.7	Association	Positive
Denmark	Ovesen et al. (30)	2004–2010	Single	RC	ICD-10 O24.4	9,014/389,606	8.2/3.9	Association	Positive
France	Cosson et al. (36)	2002–2010	Single	RC	French criteria/WHO1985	2,097/13,436	3.1/2.0	Association	Positive
	Billionnet et al. (39)	2012	All	RC	IADPSG	57,629/735,519	2.6/1.6	Association	Positive
Germany	Weschenfelder et al. (45)	2012–2016	Single	RC	IADPSG/WHO2013	614/5,175	6.8/2.7	Association	Negative
Israel	Košir Pogačnik et al. (46)	2002–1026	Single	RC	NDDG /IADPSG	10,248/226,676	2.1/1.8	Association	Negative
Japan	Shindo et al. (43)	2000–2009	Single	RC	IADPSG	503/2,789	2.0/1.8	Association	Negative
Sweden	Ostlund et al. (32)	1992–1996	Single	RC	ICD-9 648W	3,448/427,404	6.1/2.8	Association	Positive
	Fadl et al. (33)	1991–2003	Single	RC	ICD-9/ICD-10	10,525/1,249,772	5.9/2.6	Association	Positive
	Hilden et al. (31)	1998–2012	Single	RC	ICD-10 O24.4	13,057/1,252,093	3.4/1.8	Association	Positive
Uruguay	Conde-Agudelo et al. (17)	1985–1997	All	RC	ICD-10 O24.4	5,309/873,371	17.2/4.9	Association	Positive
USA	Joffe et al. (44)	1995	Single	PC	NDDG	81/3,381	12.4/7.7	Association	Negative
	Bryson et al. (38)	1992–1998	All	CC	ICD-9 648.8	Total 62,982	NR	Association	Positive

*ADA, American Diabetes Association; CC, case-control; CDA, Canadian Diabetes Association; GDM, gestational diabetes mellitus; IADPSG, International Association of Diabetes and Pregnancy Study Groups; ICD, International Classification of Diseases; NDDG, National Diabetes Data Group; NR, not reported; OGTT, oral glucose tolerance test; PC, prospective cohort; PE, preeclampsia; RC, retrospective cohort; WHO, World Health Organization*.

PE also affects the occurrence of GDM; a retrospective cohort study in Korea showed that a history of PE in first pregnancy is a risk factor for the development of GDM in subsequent pregnancies (50). However, a retrospective study in Chile suggested that the history of PE in previous pregnancies is negatively associated with the occurrence of GDM in the next pregnancy (13). Whether PE associated with the occurrence of GDM should be further confirmed in large sample studies.

## Factors Associated With Occurrence of PE in Women With GDM

### Pre Pregnancy BMI

BMI is a common index used to evaluate nutritional status, even among pregnant women. The World Health Organization (WHO) categorizes BMI into underweight, normal weight, overweight, and obesity with values <18.50, 18.50–24.99, 25.00–29.99, and ≥30.00 kg/m^2^, respectively (51). Obesity is a common risk factor for GDM and PE, an individual participant data meta-analysis of European, North American and Australian cohorts showed that obesity increased the risk of GDM by three times and the risk of PE by two times (52). Both obesity and GDM are independent associated with PE (16, 31, 32), and the combination of the two has a greater impact than either one alone (16, 31). A Sweden population-based retrospective cohort study that included the data of 13,057 women with GDM who needed treatment, analyzed the impact of pre pregnancy BMI on PE, and showed the highest risk of GDM with obesity, but without significant interaction between obesity and GDM. In addition, obesity had less effect on PE in women with GDM comparing with women without GDM, which may be the result of insulin resistance in both GDM and obesity (31). Most studies suggested that pre pregnancy BMI was independently associated with the occurrence of PE in women with GDM (23, 53–56). In a retrospective cohort study, maternal obesity, early GDM diagnosis and poor glycemic control were the three independent factors related to PE in women with GDM, of which obesity was the highest risk (56). Only one population-based retrospective study in France suggested that the incidence of PE in women with GDM was not associated with pre pregnancy BMI (36). In a randomized controlled trial evaluating metformin for GDM, obesity was not associated with PE in metformin- and/or insulin-treated women, but the incidence of PE was significantly associated with being overweight. The reason for this result may be related to the drug treatment and blood glucose control; furthermore, aspirin was not excluded as a confounding factor (57). In a prospective observational study, considering the level of blood glucose control and treatment methods, obesity was only related to PE in insulin treatment group with poor blood glucose control, but not in diet treatment group (regardless of blood glucose control) and insulin treatment group with good blood glucose control (58). Thus, the effect of pre pregnancy BMI on PE in women with GDM may be also related to blood glucose level and treatment methods.

### Gestational Weight Gain (GWG)

GWG, another commonly used indicator for nutritional status during pregnancy, is related to pregnancy complications (59, 60). In 2009, the Institute of Medicine (IOM)/National Academy of Medicine (NAM) recommended that the total weight gain during pregnancy of underweight, normal weight, overweight, and obese women according to the WHO BMI classification should respectively be 12.5–18.0, 11.5–16.0, 7.0–11.5, and 5.0–9.0 kg. The average weekly weight gain in the middle and third stages of gestation should be 0.51 (0.44–0.58), 0.42 (0.35–0.50), 0.28 (0.23–0.33), and 0.22 (0.17–0.27) kg, respectively (61). A meta-analysis reported that 30, 34, and, 37% of women with GDM had insufficient, adequate, and excessive GWG (which occurred more in pre pregnancy overweight or obese women), respectively (62). Although GWG is significantly elevated in women with GDM combined with PE (23, 53), most studies considered that the overall excess GWG had no independent correlation with the occurrence of PE (23, 36, 53, 63–65); the same result was reported for obese women with GDM (66). A recent retrospective cohort study of 1,606 women with GDM in China reported different conclusions, after adjusting for maternal age, pre pregnancy BMI, maternal education, *in vitro* fertilization, fasting, and 2 h glucose, the risk of the total excess GWG developing to PE is 2.06 times; with 2.28 and 2.17 times in the second and third trimesters, respectively (67). It has also been suggested that weight gain in early pregnancy is associated with the occurrence of PE (57). Since we were unable to determine whether pregnant women would develop GDM at the beginning of pregnancy, the management of weight after GDM diagnosis was more significant. A retrospective cohort study conducted in the US evaluated the effect of GWG on pregnancy outcome after the diagnosis of GDM; however, GWG after the diagnosis of GDM was not related to the occurrence of PE adjusted for black, pre pregnancy BMI, and chronic hypertension. However, in a logistic regression model, the weekly weight gain of pregnant patients after GDM diagnosis was evaluated as a continuous variable, after adjusting the pre pregnancy BMI, mother's age, and weekly weight gain before GDM diagnosis, the probability of PE increased by 83% for every 0.45 kg/week of weight gain (68). Recent prospective cohort study in China suggested that a unit increase in GWG level after GDM diagnosis is not related with the occurrence of PE, but in women with excessive GWG before GDM diagnosis, both adequate and excessive GWG after GDM diagnosis increased the incidence of PE (69). Both insufficient weight gain after diagnosis of GDM and total insufficient GWG are not associated with the occurrence of PE (36, 65, 67–69); however, in obese women with GDM, total insufficient GWG is negatively associated with the occurrence of PE (66). A meta-analysis of GWG and GDM pregnancy outcomes showed that excessive GWG is associated with an increased risk of pregnancy-induced hypertension, but PE was not analyzed separately (62). Further clinical studies are required to evaluate whether GWG affects PE occurrence in women with GDM, especially after the diagnosis of GDM. Furthermore, whether the recommended GWG criteria by the IOM /NAM in 2009 is applicable to women with GDM also requires further validation. A Chinese study found that among women with GDM, with weight gain within the receiver operating characteristic target, the incidence of pregnancy-induced hypertension is lower than that of women with weight gain within the IOM target (70). In an Australian study, for the GWG of women with GDM, 2 kg was subtracted from that of the IOM target, which did not improve the prognostic outcome (71).

GWG not only affects the occurrence of PE in women with GDM, but also perinatal outcomes in those complicated by PE. Although total excessive GWG is a protective factor for preterm birth, middle trimester excessive GWG is a risk factor for large gestational age and late trimester excessive GWG is a risk factor for severe PE and cesarean section (72). In conclusion, although GWG has no greater impact than pre pregnancy BMI on PE in women with GDM, it is a controllable factor during pregnancy.

### Time of GDM Occurrence

There is no consensus on the screening and diagnosis of GDM in early pregnancy. Screening is usually recommended in the first trimester or during prenatal care to exclude the presence of diabetes in high-risk women (10, 11, 73). Opinions also differ on whether early- or later-onset GDM affects PE. This may be related to the heterogeneity in diagnostic criteria for early-onset GDM and different time definitions and sample sizes. A large retrospective cohort study in Portugal including 18,518 pregnant women with GDM reported that the incidence of early-onset GDM (≤ 12 weeks) was 34.4% according to the IADPSG diagnostic criteria; there was no difference in the incidence of PE between women with early- and later-onset GDM (74). Furthermore, several studies have reported the same conclusion (55, 75–79). In early-onset GDM, metformin or insulin treatment is more needed (75, 78, 79), it is uncertain whether it will affect the occurrence of PE. However, women with early-onset GDM have more risk factors for PE, such as older age, multiple pregnancy, and higher pre pregnancy BMI (80, 81). Others suggest a significantly increased incidence of PE in women with early-onset GDM (81–84). A retrospective US cohort study of 2,596 diet-treated women with GDM shows a 2-fold incidence of PE in early diagnosis (<24 weeks) compared to women with GDM diagnosed after 24 weeks; the risk was 2.4-fold even after adjusting for maternal age, race, parity, weight, and glycemic control differences (82). In another prospective cohort study evaluating the risk of PE in women with GDM, the risk of PE in GDM diagnosed within 20 weeks of pregnancy was 8-fold, even after adjusting for pre pregnancy BMI, OGTT and control blood glucose levels (56). Whether early- or later- onset GDM affects the occurrence of PE needs to be verified by large sample prospective trials. However, for the high-risk population with GDM, early screening and active treatment may reduce the risk of PE (85, 86).

### Blood Glucose

The HAPO study showed that PE is linearly positively associated with the maternal glucose level, for every 1-standard deviation increase in OGTT blood glucose (fasting, 1 h, and 2 h), with the odds ratio of PE between 1.21 and 1.28 (29). The 5th International Symposium on Gestational Diabetes recommended that the blood glucose control criteria during pregnancy for fasting, 1 h, and 2 h blood glucose levels be <5.3, <7.8, or <6.7 mmol/L, respectively (87). The risk of PE in women with GDM increases with increasing levels of glucose impairment at diagnosis (88, 89). However, optimizing glycemic treatment can reduce the occurrence of PE (23, 90, 91), and poor glycemic control is related to the occurrence of PE (54, 56–58). Whether the blood glucose level at OGTT or the blood glucose control level can independently predict the occurrence of PE is unclear. As previously reported, blood glucose control is an independent risk factor for PE (56, 57), and OGTT blood glucose levels are not associated with the occurrence of PE (56, 57, 92). However, others reported that although optimizing blood glucose treatment can reduce the risk of PE, it is not an independent influencing factor, but the OGTT fasting blood glucose level is independent and significantly correlated with the occurrence of PE (23). Accordingly, two other studies also support the finding that OGTT blood glucose levels are an independent risk factor for the development of PE (93, 94). A Chinese retrospective cohort study reported that the blood glucose level at OGTT and after treatment of GDM did not independently predict the occurrence of PE, while the fasting blood glucose level at OGTT is an important risk factor for such (54). In conclusion, blood glucose levels should be more strictly controlled in women with severely-impaired glucose tolerance. Some prospective cohort studies and meta-analysis showed that glycosylated hemoglobin (HbA1c) ≥ 5.9% in early pregnancy significantly was associated with the risk of PE (95–98). It is controversial whether HbA1c level in the second trimester of pregnancy is related to the occurrence of PE, the secondary analysis of HAPO and a retrospective study showed that the HbA1c level during OGTT was related to the occurrence of PE (99, 100). However, two retrospective studies showed that HbA1c level in the second trimester of pregnancy was not associated with PE (54, 101). A higher HbA1c level (5.5–5.9%) within the normal range during OGTT also is an independent risk factor for preeclampsia in women with GDM in a China retrospective cohort study (102). The risk of PE in women with GDM is also related to blood glucose variability. Women with poor blood glucose monitoring compliance are more susceptible to PE than women with good compliance (103). Continuous blood glucose monitoring is helpful for detecting all postprandial blood glucose peaks and recording the impact of diet. It is conducive for the timely adjustment of the treatment plan and for reducing blood glucose variations. The incidence of PE is significantly lower than that in women whose blood glucose alone is monitored, and the mean amplitude of glycemic excursions is also an independent risk factor for PE (104). Blood glucose variability may affect the occurrence of PE by increasing oxidative stress. However, a prospective study with a small sample size showed that the glycemic variability in the third trimester of non-insulin dependent GDM was not associated with the incidence of PE (105).

### Age, Parity, and Ethnicity

Age, parity and race are uncontrollable factors, which are also related to the occurrence of PE in GDM women. More studies suggested that advanced age was not independent associated the occurrence with PE in women with GDM (23, 54, 55). Only a retrospective study considered that advanced age was an independent risk factor for PE in women with GDM (53). In a randomized controlled trial, nulliparity was independently associated with the occurrence of PE in women with GDM (57), another retrospective study reached the same conclusion (53). Other studies showed that parity was not associated with PE in women with GDM (23, 54, 55). Ethnicity also has an impact on the occurrence of PE in women with GDM. In a randomized controlled trial in New Zealand/Australia, 724 multi-ethnic subjects were included, the risk of PE in Polynesian is twice that in European/Caucasian/mixed (57). In the retrospective study in US, there was no difference in the incidence of PE among different ethnicity, including Mexican American, Caucasian and African American (53). The same conclusion was reached in the retrospective study of Fiji, that is ethnicity had no effect on the occurrence of PE in women with GDM (55).

Table 2 summarizes whether the above factors are independently associated with the occurrence of PE in women with GDM.

**Table 2:** Factors independent affecting the incidence of preeclampsia in women with gestational diabetes mellitus.

**References**	**Country**	**Study design**	**Number of GDM**	**BMI**	**eGWG**	**iGWG**	**Early GDM**	**OGTT level**	**Glucose control**	**Age**	**Parity**	**Ethnicity**
Yogev et al. (23)	USA	RC	1,813	Y	N			Y	N	N	N	
Cosson et al. (36)	France	RC	2,097	N	N	N						
Yogev et al. (53)	USA	RC	1,664	Y	N					Y	Y	N
Sun et al. (54)	China	RC	779	Y				N	N	N	N	
Osuagwu et al. (55)	Fiji	RC	255	Y			N			N	N	N
Phaloprakarn et al. (56)	Thailand	PC	813	Y			Y	N	Y			
Rowan et al. (57)	New Zealand / Australian	RCT	724	Y	Y			N	Y		Y	Y
Egan et al. (63)	Ireland	PC	543		N							
Kase et al. (64)	USA	RC	90		N							
Xie et al. (65)	Spain	RC	2,700		N	N						
Lima Ferreira et al. (66)	Portugal	PC	4,563		N	Y						
Shi et al. (67)	China	RC	1,606		Y	N						
Harper et al. (68)	USA	RC	635		Y	N						
Zheng et al. (69)	China	PC	3,126		Y	N						
Hosseini et al. (77)	Iran	PC	171				N					
Immanuel et al. (79)	New Zealand	RC	1,573				N					
Hawkins et al. (82)	USA	RC	2,596				Y					
Kalok et al. (93)	Malaysia	RC	1,105					Y				
Barden et al. (94)	Australia	PC	184					Y				

*BMI, body mass index; eGWG, excessive gestational weight gain; GDM, gestational diabetes mellitus; iGWG, insufficient gestational weight gain; N, no; OGTT, oral glucose tolerance test; PC, prospective cohort; RC, retrospective cohort; RCT, randomized controlled trial; Y, yes*.

### Effect of GDM Treatment

The first treatment of GDM is lifestyle intervention, including diet and daily activities. When hyperglycemia is evident after ≥1–2 weeks of lifestyle interventions, daily glucose testing should be continued, and pharmacological treatment should be initiated. Insulin is the most traditionally-preferred drug; oral hypoglycemic drugs, glibenclamide and metformin, are also used in some countries (2). Considering that these drugs can cross the placenta, the American Diabetes Association does not recommend them as first-line drugs for GDM (106). Two randomized controlled trials showed that intervention with GDM (including dietary recommendations, blood glucose monitoring, and insulin treatment) significantly reduced the risk of PE (90, 91), and a meta-analyses revealed similar conclusions (107).

#### Lifestyle Intervention

Lifestyle intervention mainly includes diet and exercise, the diet should contain sufficient macronutrients and micronutrients, carbohydrates with low glycemic index are recommended (2). A randomized controlled trial showed that a Mediterranean Diet, supplemented with extra-virgin olive oil and pistachios, can reduce the incidence of adverse pregnancy events of GDM, the incidence of PE in women with GDM was not different from that without GDM (108). However, omega-3 fatty acids supplementation had no effect on the incidence of PE in women with GDM (109). Inositol is considered as a food supplement, randomized controlled trials and meta-analysis believe that it can prevent the occurrence of GDM, but it cannot prevent the occurrence of pregnancy induced hypertension in high-risk groups of GDM (110–112). For women diagnosed with GDM, inositol supplementation also cannot reduce the risk of pregnancy induced hypertension/PE (113–115). Meta-analysis showed that there was a significant negative association between smoking during pregnancy and incidence of PE (116), but smoking during pregnancy did not reduce the incidence of PE in women with GDM in a retrospective cohort study (117). Moderate exercises during pregnancy are helpful to control pregnancy weight and blood glucose for women with GDM, but it has no effect on the occurrence of PE (118, 119).

#### Insulin

Although women with GDM who require insulin treatment tend to have higher blood glucose at OGTT (120–122), there was no significant difference in the incidence of PE compared to that in women on diet treatment alone (23, 39, 120–122). This may be related to the better management in the insulin treatment group (121); even the incidence of PE in women with GDM treated with insulin is consistent with those with normal glucose tolerance (123). If insulin treatment reaches the established blood glucose control level, the risk of PE in GDM with obesity is not different from that in normal weight (58).

#### Glibenclamide

For the use of glibenclamide and insulin in GDM, more comparisons were reported on blood glucose control and neonatal outcomes, and less on PE. A retrospective cohort study in California showed that the incidence of PE in a glibenclamide-treated group was twice that in an insulin-treated group, and the risk was still 2.32 times higher after adjusting for confounding factors (124). Another randomized controlled trial study found that there was no difference in the incidence of PE between glibenclamide- and insulin-treated groups (125). The same result was found even among women with GDM with significantly increased oral glucose stimulation test and fasting hyperglycemia (126).

#### Metformin

A randomized controlled trial of 733 pregnant women in 10 hospitals in New Zealand and Australia compared the pregnancy outcomes between the administration of metformin and insulin for GDM. Although the incidence of PE in the metformin-treated group was lower than that in the insulin-treated group, the difference was not statistically significant (127). In other studies, metformin also had no effect on the incidence of PE (128–131). However, the metformin-treated group had less weight gain after treatment (127, 129). No relevant data exist on whether metformin is advantageous in obese women with GDM. A recent meta-analysis evaluated the efficacy of metformin, glibenclamide, and insulin in the treatment of GDM. Metformin showed a trend of reducing PE compared with insulin, but there was no significant difference. The incidence of PE in the glibenclamide-treated group was slightly higher than that in insulin-treated group; however, there was also no significant difference (132). Metformin may prevent PE by reducing the production of anti angiogenic factors, improving endothelial dysfunction and changing cell homeostasis and energy allocation (133), it is expected to become an ideal drug for preventing PE in women with GDM.

## Effect of GDM on the Occurrence of PE in Twin Pregnancy and in Pregnant Women With PCOS

### Twin Pregnancy

With the increase in maternal age and application of assisted reproductive technology, the incidence of twin pregnancy has been increasing (134, 135). Twin pregnancy is a common risk factor of GDM and PE (1, 2), the correlation between twin pregnancy and hypertensive disease/PE was higher than that of GDM (34, 136). Population-based retrospective cohort studies across different time periods (2005–2011 and 2012–2016) in Canadians show a higher incidence of PE in twin pregnancies than in singletons, with or without GDM (35, 40). Retrospective case control studies in China and Australia also show a significantly higher incidence of PE in women with GDM with twin pregnancies than that in women with singletons (137, 138). The two population-based cohort studies in Canadians derived different conclusions on whether GDM is associated with PE in twin pregnancy. Early studies show that the risk of PE in twin pregnancy women with GDM is still 1.54-fold after adjusting for maternal age, ethnicity, parity, and prior hypertension (40). However, recent studies suggest that GDM was not associated with PE in twin pregnancy after adjusting for maternal age, smoking, parity, race, pre pregnancy BMI, and auxiliary factors (35). This difference may be related to the fact that early studies did not adjust for pre pregnancy BMI, because obesity significantly increases the risk of PE (52). The conclusions of other studies revealed inconsistencies; some suggested that the incidence of PE in twin pregnant women with GDM was significantly higher than that without GDM (135, 138, 139), and GDM was independent associated with PE in twin pregnancy (135, 139), while others suggested no significant difference between the two groups (140, 141). Whether GDM is independent associated with PE in twin pregnancy requires further validation; overall, GDM has less impact on PE in twin pregnancy than in singleton pregnancy (35, 40, 135).

### PCOS

Women with PCOS have increased insulin resistance and hyperandrogenemia (142, 143). PCOS increases the incidence of GDM and PE, and the results are independent of obesity and assisted reproductive technology (144–146). A meta-analysis showed that pregnant women with PCOS had a 2.89- and 1.87-fold risk of GDM and PE, respectively (145). The incidence of PE is significantly higher in women with GDM combined with PCOS than in those without PCOS (147–150). The risk was 2-3-fold after adjusting for factors such as age, pre pregnancy BMI, and parity (148–150). Only one study believes that there was no correlation between PCOS and PE in women with GDM after adjusting for confounding factors (147). A Chinese prospective study found no difference in the incidence of PE between women with GDM with PCOS and those without PCOS, although the result was affected by factors such as small sample size and early intervention (151). Pregnant women with PCOS combined with GDM tend to be older and have higher pre pregnancy BMI (152–154). It is necessary to determine whether GDM associated with PE in pregnant women with PCOS. A prospective, double-blind, multicenter trial including 228 pregnant women with PCOS in Norway revealed that there was no statistical correlation between early GDM and PE occurrence (152). Similarly, two other studies were added to the earlier one, which increased the number of pregnant women with PCOS to 722. The results still show that GDM did not increase the incidence of PE in pregnant women with PCOS, regardless of whether GDM occurred early or later (153). Another prospective study in China found that the incidence of PE in PCOS pregnant women with GDM is significantly higher than that in pregnant women without GDM but did not analyze whether it was independent associated with PE (154). Thus, the effect of GDM on PE in pregnant women with PCOS is less than the effect of PCOS on PE in women with GDM.

## Mechanism and Predictive Markers

**Figure 1:**
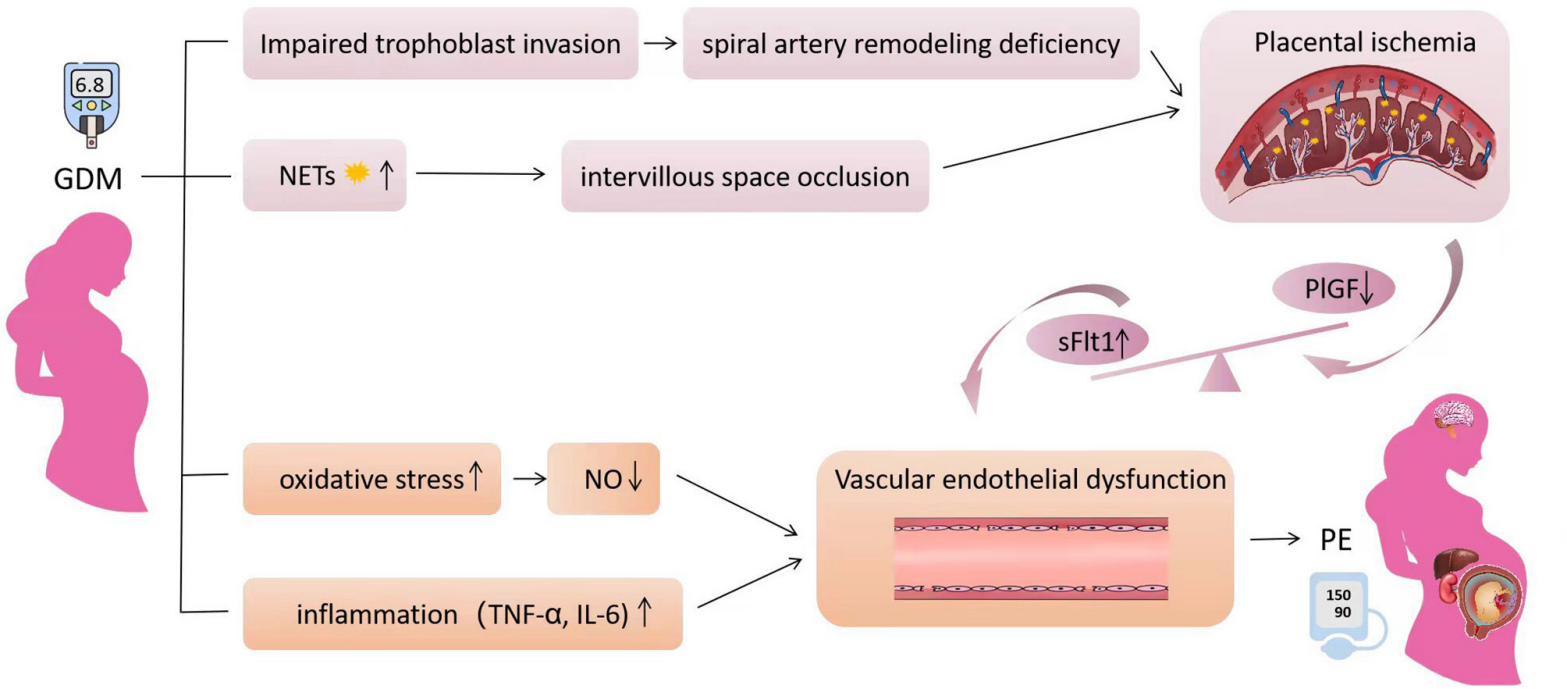
The mechanism of gestational diabetes mellitus affecting the occurrence of preeclampsia. Hyperglycemia inhibits trophoblast migration and invasion by inducing trophoblast inflammation and autophagy, which can lead to uterine spiral artery remodeling deficiency. Neutrophils in GDM are over-activated and release excessive neutrophil extracellular traps resulting intervillous space occlusion. The two factors cause placental ischemia and hypoxia, resulting in the imbalance of anti-angiogenic factors, which can lead to vascular endothelial injury. In addition, increased oxidative stress in GDM leads to decreased nitric oxide synthesis and activity, resulting in vasodilation dysfunction. The increased inflammatory factors further aggravate the vascular endothelial injury. The clinical symptoms of preeclampsia eventually appeared, including hypertension and multiple organ injury. Obesity exaggerates all pathways affecting PE.

Although GDM is associated with PE, the exact mechanism underlying the two disease associations is unclear. The pathophysiological process of PE involves two stages, early insufficient trophoblast invasion leads to incomplete spiral artery remodeling, which causes placental ischaemia and oxidative stress. The diseased placenta progressively secretes elevated amounts of anti-angiogenic factors [soluble fms-like tyrosine kinase-1 (sFlt1) and soluble endoglin] that cause maternal inflammation and vascular endothelial dysfunction, and finally lead to systemic diseases (1, 155). Hyperglycemia can induce trophoblast inflammation and autophagy, inhibit trophoblast migration and invasion (156–158). Neutrophils in GDM are over-activated and release excessive neutrophil extracellular traps (NETs) (159, 160). Excessive NETs hinder the blood circulation in the villous space, resulting in placental ischemia, which is related to the occurrence of PE (161–163). Oxidative stress increases in women with GDM (164–166), hyperglycemia can induce oxidative stress through a variety of ways, including the formation of advanced glycation end products (AGEs) (166). The production of reactive oxygen species increases during oxidative stress, resulting in a decrease in circulating nitric oxide (NO) level and bioavailability (166), which leads to vasodilation dysfunction. AGEs are significantly increased in women with GDM (167), and can promote the occurrence of PE by inducing oxidative stress and inflammation (168–170). Moreover, the pro-inflammatory cytokines serum tumor necrosis factor-α(TNF-α) and Interleukin-6 (IL-6) have been found increased in the circulation of women with GDM (171, 172), which are associated with endothelial dysfunction (172), and also increased in women with PE (173, 174). Some studies suggest that TNF-α, IL-6, and C-reactive protein (CRP) are independent risk factors for PE in women with GDM (94, 175, 176), and others suggest that in addition to the increased level of CRP, the imbalance of Interleukin-17 /Interleukin-35 may also be involved in the pathogenesis of GDM complicated with PE (177). Genetic variants are also associated with PE in women with GDM, the MIR146Ars2910164CC genotype, HNF1αgene p. I27L TT genotype, and ACE I / D polymorphism DD genotype was significantly higher in women with GDM complicated with PE (178–180). Obesity is the main influencing factor of PE in women with GDM in this paper. There are many of same pathophysiological changes between obesity and GDM, but obesity was concluded to be associated with greater oxidative stress and inflammation including the imbalance of fat factors (181), which are related to the occurrence of PE. Hyperinsulinemia and insulin resistance caused by obesity before pregnancy are related to the migration of cytotrophoblast and the reduction of uterine spiral artery remodeling, which is more likely to lead to placental ischemia (182). The mechanism of gestational diabetes mellitus affecting the occurrence of preeclampsia is shown in Figure 1.

Multiple biochemical markers have been studied to predict the occurrence of GDM and PE, and CRP, TNF-α, IL-6, and B-type natriuretic peptides are common predictive markers (183, 184), but none are used as practical clinical markers. Serum sFlt1 / placental growth factor (PlGF) is a valid marker for predicting and diagnosing PE (185). It is also significantly elevated in the blood of women with GDM complicated with PE (186). However, whether it can early identify the risk of PE in women with GDM needs further research. In conclusion, there are no practical markers to predict the occurrence of PE in women with GDM, and we need to explore the pathophysiology of GDM and PE further.

## Conclusion

In most studies, GDM is independently associated with PE in singleton pregnancy, and pre pregnancy BMI and blood glucose levels are closely related with the occurrence of PE. Therefore, optimizing the treatment and management of GDM can reduce the incidence of PE. Oral hypoglycemic drugs, including metformin and glibenclamide, showed no significant difference in the occurrence of PE compared with insulin, despite a decreasing trend for metformin. The effects of GWG on PE, especially after the diagnosis of GDM and early-onset GDM, are controversial, and thus warrant further prospective studies. Twin pregnancy and PCOS significantly increased the occurrence of PE in women with GDM. However, GDM has less effect on PE in twin pregnancy and pregnant women with PCOS. The prevalence of GDM is significantly increased, which also increases the incidence of PE. Therefore, identifying the controllable factors affecting PE of GDM is important for improving pregnancy outcomes. GDM may affecting the occurrence of PE by inducing placental ischemia, increasing oxidative stress and inflammation. Understanding the pathophysiological mechanism of GDM affecting the occurrence of PE is helpful to find effective markers and preventive measures, which needs further studies.

## Author Contributions

YY collected material and wrote the first draft. NW contributed to design of the study and provided critical feedback. All authors contributed to the article and agree to be accountable for the content of the work.

## Funding

This study was funded by the National Natural Science Foundation of China (No. 81700706), the 345 Talent Project of ShengJing Hospital, the Clinical Research Project of Liaoning Diabetes Medical Nutrition Prevention Society (No. LNSTNBYXYYFZXH-RS01B), Natural Science Foundation of Liaoning Province (No. 2021-MS-182), the Science Foundation of Liaoning Education Department (No. LK201603), and the Virtual Simulation Experiment Teaching Project of China Medical University (No.2020-47).

## Conflict of Interest

The authors declare that the research was conducted in the absence of any commercial or financial relationships that could be construed as a potential conflict of interest.

## Publisher's Note

All claims expressed in this article are solely those of the authors and do not necessarily represent those of their affiliated organizations, or those of the publisher, the editors and the reviewers. Any product that may be evaluated in this article, or claim that may be made by its manufacturer, is not guaranteed or endorsed by the publisher.

## Abbreviations

AGEs: advanced glycation end products
BMI: body mass index
CRP: C-reactive protein
GDM: gestational diabetes mellitus
GWG: gestational weight gain
HAPO: Hyperglycemia and Adverse Pregnancy Outcome
HbA1c: glycosylated hemoglobin
IADPSG: International Association of Diabetes and Pregnancy Study Groups
IL-6: interleukin-6
IOM: Institute of Medicine
NAM: National Academy of Medicine
NETs: sneutrophil extracellular traps
NO: nitric oxide
OGTT: oral glucose tolerance test
PCOS: polycystic ovary syndrome
PE: preeclampsia
PlGF: placental growth factor
sFlt1: soluble fms-like tyrosine kinase-1
TNF-α: tumor necrosis factor
WHO: World Health Organization.
